# Evaluation of Pathological Association between Stroke-Related QTL and Salt-Induced Renal Injury in Stroke-Prone Spontaneously Hypertensive Rat

**DOI:** 10.1155/2019/5049746

**Published:** 2019-01-16

**Authors:** Mohammad Farhadur Reza, Davis Ngarashi, Masamichi Koike, Masaki Misumi, Hiroki Ohara, Toru Nabika

**Affiliations:** ^1^Department of Functional Pathology, Shimane University Faculty of Medicine, Izumo, Japan; ^2^Department of Oncology, Shimane University Faculty of Medicine, Izumo, Japan

## Abstract

The stroke-prone spontaneously hypertensive rat (SHRSP) suffers from severe hypertension and hypertensive organ damage such as cerebral stroke and kidney injury under salt-loading. By a quantitative trait locus (QTL) analysis between SHRSP and SHR (the stroke-resistant parental strain of SHRSP), two major QTLs for stroke susceptibility were identified on chromosomes 1 and 18 of SHRSP, which were confirmed in congenic strains constructed between SHRSP and SHR. As the progression of renal dysfunction was suggested to be one of the key factors inducing stroke in SHRSP, we examined effects of the stroke-related QTLs on kidney injury using two congenic strains harboring either of SHRSP-derived fragments of chromosomes 1 and 18 in the SHR genome. The congenic strains were challenged with 1% NaCl solution for 4 weeks; measurement of systolic blood pressure and urinary isoprostane level (a marker for oxidative stress) and evaluation of renal injury by quantification of genetic marker expression and histological examination were performed. We found that the congenic rats with SHRSP-derived fragment of chromosome 18 showed more severe renal damage with higher expression of* Col1α-1* (a genetic marker for renal fibrosis) and higher urinary isoprostane level. In contrast, the fragment of chromosome 1 from SHRSP did not give such effects on SHR. Blood pressure was not greater in either of the congenic strains when compared with SHR. We concluded that the QTL region on chromosome 18 might deteriorate salt-induced renal injury in SHR through a blood pressure-independent mechanism.

## 1. Introduction 

The stroke-prone spontaneously hypertensive rat (SHRSP) has been characterized as a good genetic model for severe hypertension and hypertensive organ damage such as cerebral hemorrhage [1–4]. It was therefore suggested that investigation of genetic mechanisms of stroke susceptibility in SHRSP provided us with important clues to understand genetic susceptibility to stroke in humans, which would be useful in its prevention and therapeutics [5]. In this context, several genetic studies were performed and identified quantitative trait locus (QTL) responsible for stroke occurrence [6–8]. We also identified two major QTLs for stroke on chromosomes (chr) 1 and 18 in SHRSP and confirmed their effects in reciprocal congenic strains constructed between SHR and SHRSP [9]; in brief, the congenic strains having the SHRSP-derived QTL fragments of chr_1 or 18 on SHR background showed a shorter stroke-latency when compared with SHR [9]. Of interest, these congenic strains did not show significant difference in blood pressure when compared with SHR, indicating that the greater susceptibility to stroke in these strains was blood pressure independent [9].

On the other hand, it was suggested that SHRSP suffered from severe renal damage under salt-loading when compared with SHR [3, 10, 11]. Further, several groups identified QTLs for salt-induced renal damage on chr_1 of rats, which were the vicinity of the QTL for stroke [6, 12]. If susceptibility to renal injury is influenced by the QTLs for stroke on chr_1 and/or 18, it may be useful as a clue to identify the genes responsible for stroke, and to understand the functions of those genes.

In this context, we compared salt-induced renal injury among two congenic strains and the parental strain (i.e., SHR) in this study to examine whether the QTLs on chr_1 and 18 affected renal injury. In addition, possible relevance between renal injury and the stroke susceptibility was discussed.

## 2. Materials and Methods

### 2.1. Animal Procedure

Two congenic strains for the QTLs on chr_1 and 18 [SHR.SHRSP-(*D1Rat93-D1Rat269*)/Izm and SHR.SHRSP-(*D18Rat73-D18Rat11*)/Izm, respectively] were employed in this study (abbreviated as Rp1.0 and Rp18.0, respectively). In Rp1.0 and 18.0, a chromosomal fragment of chr_1 and 18 of SHRSP/Izm was introgressed, respectively, into SHR/Izm [9]. SHR/Izm was used as the control strain. Six male rats at 12 weeks of age of each strain were used in the experiments and all rats were fed stroke permissive Japanese diet. SHR/Izm were provided by the Disease Model Cooperative Research Association (Kyoto, Japan).

After the measurement of blood pressure (BP) and body weight (BW) at 12 weeks of age, salt-loading was then started by feeding them with 1% salt water. BP and BW were monitored every week during 4 weeks of the experimental period. Urine samples were collected for 24h in metabolic cages every 2 weeks. Urine samples were centrifuged at 2000 rpm for 10 min at 4°C and the supernatants were stored at -20°C until further biochemical analysis. BP measurement was done using the tail-cuff method (BP-98A; Softron Corp., Tokyo, Japan). Each rat was acclimatized at 37°C for 10 min before BP measurement. Five consecutive readings were recorded and averaged to represent BP of an individual rat.

At the end of the experiment, each rat was deeply anesthetized in isoflurane inhalation-chamber (2% with 300~400ml/min flow rate) and perfused with ice-cold 0.9% saline solution for organ collection. The left kidney was stored in 10% formalin for histological analysis and the right kidney was dissected, frozen in liquid nitrogen, and kept at -80°C for RNA extraction. The study protocol was approved by the local ethical committee of animal research in Shimane University.

### 2.2. Biochemical Measurements

Urinary 15-isoprostane-F2t (IsoP) excretion level was measured to estimate oxidative stress [13] using an ELISA kit (JaICA, Nikken SEIL Co., Ltd.). Urinary protein level was determined in 24h urine samples as well with the protein assay BCA kit (Wako Pure Chemical Industries Ltd., Japan) [14]. The measurements were performed according to the manufacturer's protocol.

### 2.3. Renal Histopathology

For histological evaluation of renal damage, haematoxylin-eosin (HE) and Azan staining were performed on histological sections of left kidney. On HE-stained sections, glomeruli were categorized into three groups according to the severity of glomerulosclerosis (Figures 2(a)–2(c)). About 300 glomeruli were examined on each rat (about 1800 glomeruli were examined on 6 rats from each strain) and a ratio of partially + completely sclerotic and intact glomeruli was compared among the strains using *χ*2 test. The same results were obtained when we employed only completely sclerotic glomeruli instead of partially + completely sclerotic glomeruli (data not shown). On Azan staining, area of fibrotic regions (regions stained blue on Azan staining, see Figures 2(d) and 2(e)) was measured on digital images of the section using NIH Image J (ver1.8.0). A relative fibrotic area (%) was calculated as fibrotic area/total area x 100. Relative fibrotic area was compared among the strains using Student's t-test with Bonferroni's correction.

### 2.4. Gene Expression

Gene expression of* Col1α-1*,* Tgf- β*, *α-Sma *( markers for fibrosis), and* Kim-1*( a marker for tubular injury) was determined in the kidney by quantitative RT-PCR as described previously [15, 16]. The primers used are as follows: *α-Sma*: GAGATCTCACCGACTACCTCATGA (forward), TCATTTTCAAAGTCCAGAGCGACA (reverse),* Tgf- β*: ATCCATGACATGAACCGACCCT (forward), GCCGTACACAGCAGTTCTTCTC (reverse),* Col1α -1*: ACATGTTCAGCTTTGTGGACCTC (forward), TCAGGTTTCCACGTCTCACCA (reverse),* Kim-1*: GGAGCAGCGGTCGATACAACATA (forward), TCTCCACTCGGCAACAATACAGAC (reverse). The PCR condition was as follows: 1 cycle of 95°C for 30s, followed by 40 cycles of 95°C for 30s and 60°C for 30s (Step One Plus Real Time PCR System, Thermo Fisher Scientific, Waltham, MA). Relative amount of mRNA was calculated against *β*–actin as a control.

### 2.5. Statistical Analysis

All the data are presented as mean ± SD. Statistical significance was tested either by *χ*2 test or by Student's t-test. In case of multiple comparisons, significant levels were adjusted by Bonferroni's correction. Difference was thought to be significant when p<0.05 (comparison between 2 groups) or p<0.017 (comparison among 3 groups).

## 3. Results

### 3.1. Blood Pressure under Salt-Loading

**Figure 1:**
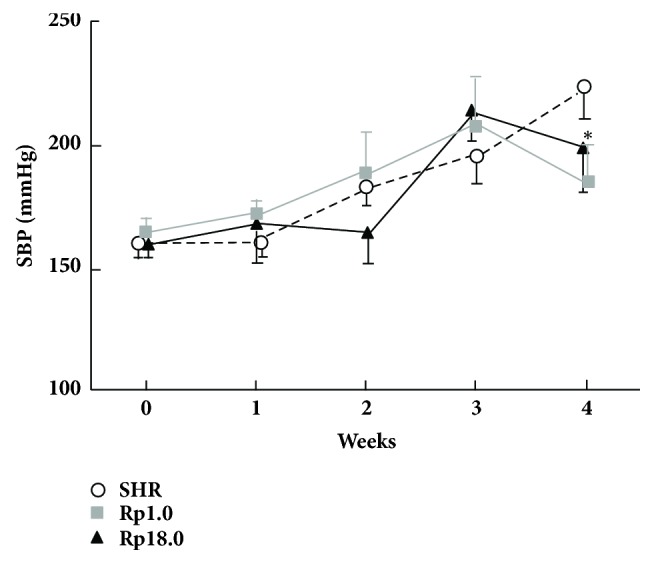
Effect of salt-loading on blood pressure. Six rats of each strain were used. ^*∗*^p<0.001* vs* control SHR (Student's t-test), which was significant after Bonferroni's correction. Data are presented as mean ± SD.

Baseline BP at 12 weeks of age was not significantly different among the strains (Figure 1). During salt-loading, BP increased gradually in all the three strains. At 4 weeks of salt-loading, BP of Rp1.0 was significantly lower than that of SHR.

### 3.2. Renal Injury Induced by Salt-Loading

**Figure 2:**
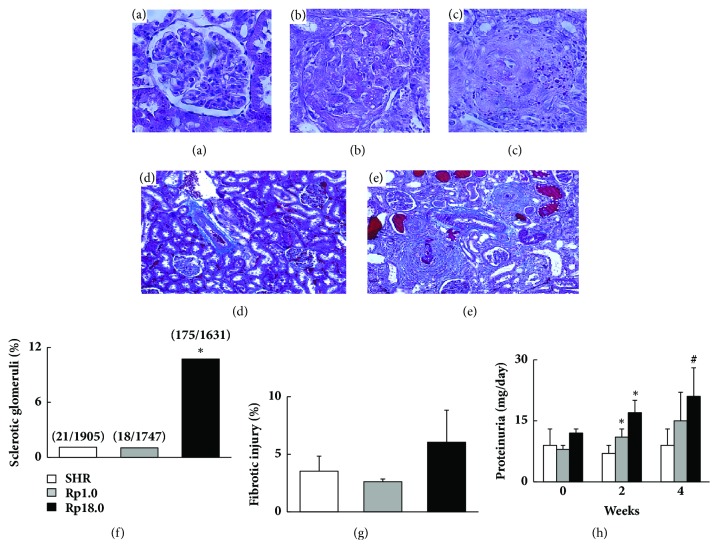
Comparison of severity of renal damages among the three strains. (a–c) Typical morphology of glomerulus with no (a), partially (b), and completely (c) sclerotic change, respectively. Photos were taken at 20X. (d, e) Typical microscopic appearance from SHR (d) and Rp18.0 (e) by Azan staining after 4 weeks of salt-loading. Photos were taken at 10X. (f-g) Prevalence of sclerotic glomeruli (f) and relative fibrotic area (g) were compared among the three strains. ^*∗*^Significantly different from both SHR and Rp1.0 after Bonferroni's correction (by the *χ*2 test). (h) Urinary protein excretion for 24h at 0, 2, and 4 weeks of salt-loading (n=6). ^*∗*^p<0.001 and ^#^p<0.01* vs* control SHR by Student's t-test, which were significant after Bonferroni's correction. Data are presented as mean ± SD.

Histopathological assessment of glomerulosclerosis showed that the number of sclerotic glomeruli was significantly greater in Rp18.0 when compared with Rp1.0 and SHR (Figure 2(f)). In accordance with it, fibrotic area tended to be increased in Rp18.0 though it did not reach a significant level (Figure 2(g)). Urinary protein level did not differ among the three strains before salt-loading as indicated (Figure 2(h)). During salt-loading, however, urinary protein excretion was increased in the two congenic strains whereas no significant increase was observed in SHR (Figure 2(h)).

### 3.3. Evaluation of Gene Expression in the Kidney under Salt-Loading

**Figure 3:**
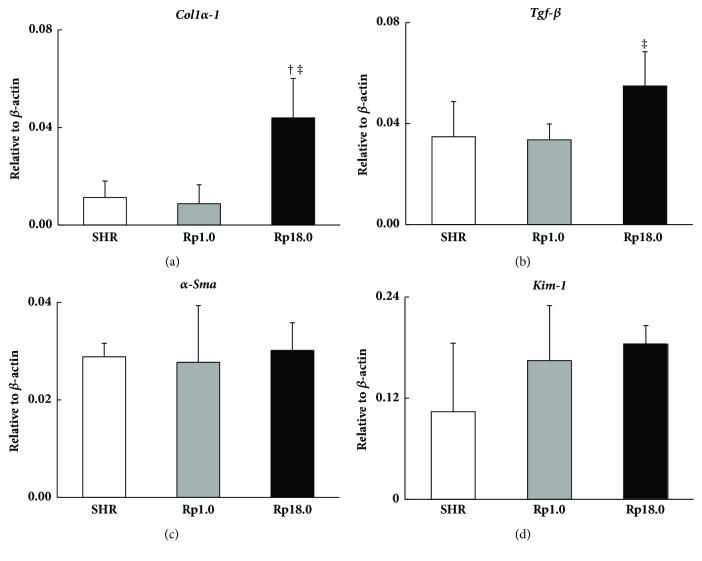
Expression of genetic markers for renal damage. (a–d) Gene expression of* Col1α-1* (a),* Tgf- β* (b), *α-Sma* (c), and* Kim-1*(d), respectively, in the kidney after 4 weeks of salt-loading (n=5 in each group). Gene expression was represented as relative amount against *β*–actin mRNA. †, ‡: significantly different from SHR (†) and Rp1.0 (‡), respectively, after Bonferroni's correction. Data are presented as mean ± SD.

We evaluated expression of genes that are biomarkers of renal fibrosis and tubular damage [17–19]. As shown in Figure 3,* Col1α-1* expression was significantly greater (p=0.003) and* Tgf- β* expression tended to be greater (p=0.049) in Rp18.0 when compared with SHR (under Bonferroni's correction). The expression of both genes was significantly different between the two congenic strains (p=0.002 and 0.012 for* Col1α-1* and* Tgf- β*, respectively). No significant difference was observed in *α-Sma* or in* Kim-1* expression among the three strains.

**Figure 4:**
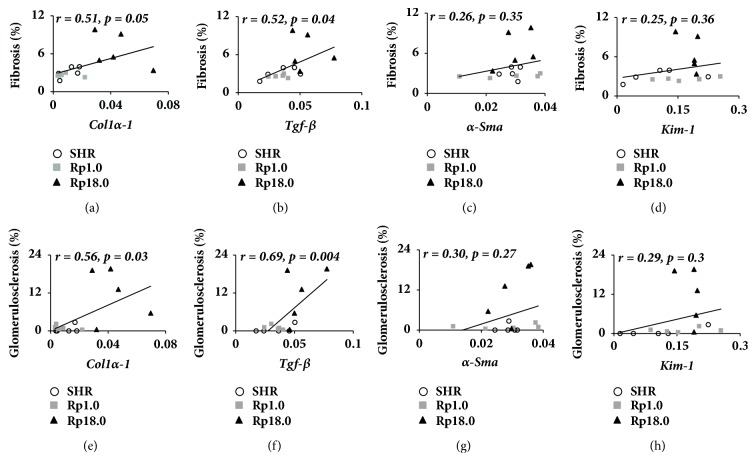
Correlation of mRNA expression with fibrosis and glomerulosclerosis. (a–d) Correlation between fibrotic area (%) and* Col1α-1* (a),* Tgf- β* (b), *α-Sma* (c), and* Kim-1 *(d) expression. (e–h) Correlation between the prevalence of glomerulosclerosis (%) and the genetic marker expressions. Pearson's r is indicated with p values (n=5 in each group).

Correlation of the gene expressions and severity of renal fibrosis and glomerulosclerosis were examined in Figure 4. The results indicated that while* Col1α-1* and* Tgf- β* expressions were correlated significantly with glomerulosclerosis and renal fibrosis, expressions of the other genes were not.

### 3.4. Salt-Loading Induced Oxidative Stress

**Figure 5:**
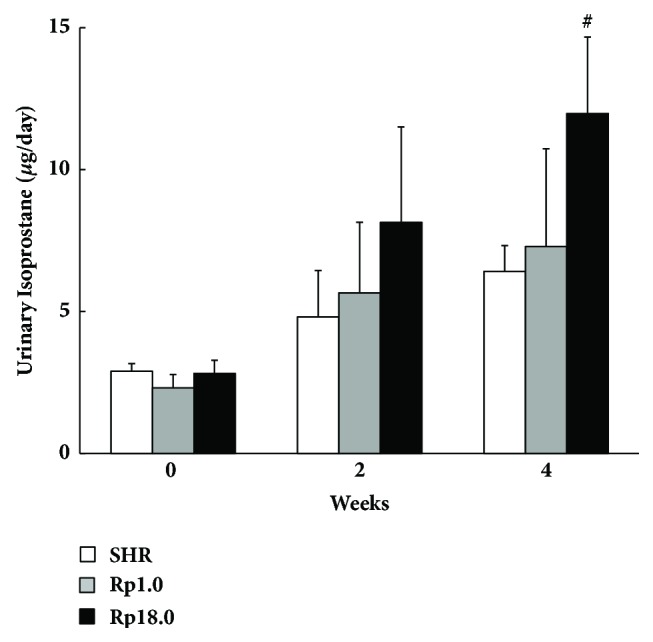
Urinary isoprostane level under salt-loading. Isoprostane was quantified in 24h urine collected after 0, 2, and 4 weeks of salt-loading (n=6 in each group). ^#^p<0.01* vs* control SHR by Student's t-test, which was significant after Bonferroni's correction. Data are presented as mean ± SD.

It was pointed out that oxidative stress plays a key role in salt-induced renal damage [20]. We therefore measured urinary isoprostane, a sensitive marker of oxidative stress* in vivo*. At the baseline, isoprostane did not significantly differ among the strains (Figure 5). During salt-loading, isoprostane was increased in all the three strains and in contrast to the baseline status, Rp18.0 showed significantly greater level of urinary isoprostane when compared with SHR after 4 weeks of salt-loading. No significant difference was observed between SHR and Rp1.0.

## 4. Discussion

In this study, we found that the congenic strain Rp18.0 was more susceptible to salt-induced renal damage than was the other congenic strain Rp1.0. It seems that the susceptibility was independent of BP as no significant difference of BP was observed between the two congenic strains. Since the previous study pointed out that the effect of chr18 QTL on stroke was BP independent [9], it is attractive to hypothesize that the same gene(s) in the QTL region contributed to both renal damage and stroke in Rp18.0. In this regard, it is of interest that several reports indicated that vasculature in SHRSP was more vulnerable to hypertensive insult [2, 3, 11].

Several different interpretations are possible for the relation between cerebral stroke and hypertensive renal damage; the renal damage may causally relate to cerebral stroke, or may just be a bystander (in another word, risk genes for stroke may have pleiotropic effects on the kidney). We can even assume another gene (or genes) for renal damage located in the same QTL region. When causal roles of renal damage are considered, a putative mechanism is acceleration of hypertension due to renal damage. However, as shown in Figure 1, BP did not become higher in Rp18.0 during salt-loading. Further, despite the fact that the difference was statistically significant, the prevalence of glomerulosclerosis and the relative area of renal fibrosis were still modest even in Rp18.0 (around 10 and 5%, respectively, see Figures 2(f) and 2(g)). This observation suggested that renal injury observed in Rp18.0 was not likely to have direct causal relationship with greater incidence of cerebral stroke in this strain.

Another hypothesis is that the QTL on chr_18 affected both stroke and renal damage as parallel events. Salt intake inhibits the release of renin from the juxtaglomerular apparatus that results in the depletion of angiotensin II (AngII) level. In contrast, some studies showed that salt stimulated the intrarenal local renin-angiotensin system (RAS) that might contribute to the regulation of renal NADPH oxidase activity [20]. NADPH oxidases facilitate the generation of reactive oxygen species (ROS) in mesangial cells in glomeruli [21] and epithelial cells in thick ascending limbs [22] which might deteriorate glomerulosclerosis and renal fibrosis [23]. As the local RAS was identified in the brain as well [24], overproduction of ROS through activation of the local RAS might contribute simultaneously to stroke [25]. In this study, a greater level isoprostane was indeed shown in Rp18.0 only under the salt-loaded status. This suggested that a higher level of oxidative stress was actually induced under salt-loading in this strain (see Figure 5).

Several studies were done to investigate molecular mechanisms of how oxidative stress influenced renal injury and stroke; in the kidney, oxidative stress (mostly generated in renal mesangial cells) was shown to activate the protein kinase C (PKC) and the mitogen-activated protein kinases (MAPK), which then promoted the translocation of transcription factors such as NF-*κ*B and AP-1 into the nucleus that eventually facilitated expression of the gene of extra cellular matrix proteins [26]. On the other hand, in the brain, oxidative stress was shown to lead activation of the extracellular signal-regulated kinases 1/2 and the N-methyl-D-aspartate receptor that facilitated Ca^2+^ influx. It then activated the cytosolic phospholipase A_2*α*_ (PLA_2*α*_) through MAPK and PKC activation [27]. The cytosolic PLA_2*α*_ enhanced production of arachidonic acid and conjugated dienic hydroperoxides that was decomposed into aldehydes, e.g., 4-hydroxynonenal which was toxic to neurons [28].

Future studies should focus on pathophysiological significance of increased oxidative stress in Rp18.0 and identify the responsible gene (or genes) for this phenomenon in the chr18 QTL region.

We had discrepancy among the expression of marker genes in the kidney; while* Col1α-1* and* Tgf- β* expression was significantly greater or tended to be greater in Rp18.0, *α-Sma* and* Kim-1* did not show a difference between Rp18.0 and the other two strains (see Figure 3). When correlation between the mRNA expression and renal fibrotic area/glomerulosclerosis was examined, we found that *α-Sma* showed no significant correlation with renal fibrotic area or with glomerulosclerosis while* Col1α-1* and* Tgf- β* did in the present study (see Figure 4). One possible reason for this discrepancy was that the expression was examined at one point, i.e., after 4 weeks of salt-loading. *α-Sma*, despite that it is a marker for fibrosis, might not be active at this point. In the meantime,* Kim-1* is known to be a marker for tubular injury. Therefore, tubular injury might not be a major player in the pathology of renal damage in the congenic strains studied here.

Although some classical parameters for renal damage, i.e., histological changes quantified by microscopic observation and urinary protein excretion, supported more advanced renal injury in Rp18.0, it may be useful to add other marker genes to obtain further support; the Bcl-3 and the urinary lipocalin-type prostaglandin D synthase were recently found to be sensitive markers for renal damage in several different diseases [29, 30], and urinary cystatin C is another established marker for renal function [31].

## 5. Conclusion

We showed that Rp18.0 suffered from more advanced renal injury under salt-loading when compared with SHR. As Rp18.0 was shown to be more susceptible to cerebral stroke in the previous study [9], this observation raised a possibility that the gene(s) in the QTL on chr_18 might induce both cerebral stroke and renal injury. We further showed that oxidative stress was significantly greater in Rp18.0, implying that oxidative stress played a pivotal role in the pathological changes observed in this congenic strain. The mechanisms of how increased oxidative stress promoted cerebral and renal injury observed in Rp18.0 is to be elucidated in a future study.

## Data Availability

The data and materials supporting the conclusions of this article are included within the article.
